# Effect of Cultural Conditions on Protease Production by a Thermophilic *Geobacillus thermoglucosidasius* SKF4 Isolated from Sungai Klah Hot Spring Park, Malaysia

**DOI:** 10.3390/molecules25112609

**Published:** 2020-06-04

**Authors:** Allison D. Suleiman, Nor’Aini Abdul Rahman, Hidayat Mohd Yusof, Fairolniza Mohd Shariff, Nur Adeela Yasid

**Affiliations:** 1 Department of Bioprocess Technology, Faculty of Biotechnology and Biomolecular Sciences, Universiti Putra, Malaysia, Serdang Selangor 43400, Malaysia; allisonsuleiman@yahoo.com (A.D.S.); hidayatmy@gmail.com (H.M.Y.); 2 Department of Food Science and Technology, School of Agriculture and Agricultural Technology, Moddibo Adama University of Technology, Yola 640230, Nigeria; 3 Department of Microbiology, Faculty of Biotechnology and Biomolecular Sciences, Universiti Putra Malaysia, Serdang Selangor 43400, Malaysia; fairolniza@upm.edu.my; 4 Department of Biochemistry, Faculty of Biotechnology and Biomolecular Sciences, Universiti Putra, Malaysia, Serdang Selangor 43400, Malaysia; adeela@upm.edu.my

**Keywords:** protease, *Geobacillus thermoglucosidasius* SKF4, hot spring, Malaysia

## Abstract

Major progress in the fields of agriculture, industry, and biotechnology over the years has influenced the quest for a potent microorganism with favorable properties to be used in scientific research and industry. This study intended to isolate a new thermophilic-protease-producing bacterium and evaluate its growth and protease production under cultural conditions. Protease producing bacteria were successfully isolated from Sungai Klah Hot Spring Park in Perak, Malaysia, and coded as SKF4; they were promising protease producers. Based on microscopic, morphological, and 16S rRNA gene analysis, isolate SKF4 was identified as *Geobacillus thermoglucosidasius* SKF4. The process of isolating SKF4 to grow and produce proteases under different cultural conditions, including temperature, pH, NaCl concentration, carbon and nitrogen sources, and incubation time, was explored. The optimum cultural conditions observed for growth and protease production were at 60 to 65 °C of temperature, pH 7 to 8, and under 1% NaCl concentration. Further, the use of casein and yeast extract as the nitrogen sources, and sucrose and fructose as the carbon sources enhanced the growth and protease production of isolate SKF4. Meanwhile, isolate SKF4 reached maximum growth and protease production at 24 h of incubation time. The results of this study revealed a new potent strain of thermophilic bacterium isolated from Sungai Klah Hot Spring Park in Perak, Malaysia for the first time. The high production of thermostable protease enzyme by *G. thermoglucosidasius* SKF4 highlighted the promising properties of this bacterium for industrial and biotechnological applications.

## 1. Introduction

Proteolytic enzymes represent one of the major classes of enzymes with commercial importance that are utilized for industrial purposes. Proteases or peptidases are a class of hydrolytic enzymes involved in hydrolyzing the peptide bonds in proteins and catalyze the synthesis of peptides in the presence of an organic solvent [1,2]. Rao et al. [3] described the protease enzyme as a hydrolytic enzyme that increases the rate of the splitting of peptides links in other proteins. Based on the range of pH in which they function, proteases or peptidases can be classified as acidic, alkaline, or neutral, and can be divided into cysteine, serine, and aspartic proteases, and metalloproteases, based on the functional groups present around their active sites [2,3,4].

Proteases have a key function regarding industrial enzymes in areas such as domestic, industrial, the processing of leather, bioremediation, nutraceutical purposes, healthcare products, development of a kit for diagnosis, and the incrementation of the value in the production for medical purposes [5]. In 2009, an estimated value of $220 billion of industrial enzymes was sold worldwide [5]. Thermophilic microorganisms devised adaptation towards growing and surviving in extreme temperatures. They can be divided into the following classes: moderately thermophilic, extremely thermophilic, and hyperthermophilic [6,7]. Therefore, the organisms are capable of being isolated from high temperature terrestrial as well as marine habitats such as volcanic and geothermal escape systems [7]. In recent times, Nazina et al. [8] arranged thermophilic bacteria bacilli that are Gram-positive with rod shape cells and formed endospores to be known as genus *Geobacillus*. They have a broad distribution and are less difficult to be isolated from the diverse natural environment [8,9]. Their thermostable gene products have been increasingly demanded by industries [10,11]. Therefore, the study of diversity and phylogenetic relationships within the genus of a new bacterium is not only a matter of significant taxonomy, but is also essential so that its biotechnological potential can be completely exploited.

*Geobacillus* represents the most common aerobic organisms isolated from hot spring samples. This genus was originally classified under *Bacillus*; however, the bacteria were regrouped into a distinct genus [12]. *Geobacillus* species have been isolated from several environments, such as high-temperature oil fields, a corroded pipeline in a very deep well [13], an African hot spring [8], a Russian hot spring, and a marine vent [14]. They are obligate thermophilic organisms that can grow in temperatures ranging from 37 to 75 °C and grow optimally between 55 and 65 °C. In this regard, most members of the genus are found in warm biotopes, such as most soil environments, heaps of compost, oil fields, and geothermal zones [12,15]. Unexpectedly, these organisms can also be discovered in low-temperature habitats, such as soil that has never been subjected to extreme temperatures, or at the lowest part of the sea [12,16].

One of the natural environments wherein thermophilic bacteria can be widely found is the hot spring. Hot springs are where hot geothermal water comes out from the ground. They can be found in every part of the world, including underneath the oceans and seas [15]. Studies based on molecular phylogenetic approaches using 16S rRNA to investigate different bacteria found in the hot spring have been undertaken lately [15]. Thermophilic bacteria have been characterized by phenotypic and genotypic means from numerous geothermal regions of the world [15]. Saxena et al. [17] and Sahay et al. [18] described thermal springs as being a very strong habitat for a potential microorganism with unique enzymes, molecules, and genes, which could advance applications in medicine, agriculture, and industry.

*Geobacillus thermoglucosidasius* (also known as *G. thermoglucosidans*) are endospore-forming Gram-positive, facultative, and thermophilic anaerobes. They are organisms that have the potential to produce chemicals and to be used for bioremediation [19,20]. Naturally, representatives of *Geobacillus* species are isolated in different environments such as volcanic springs, hydrothermal openings, oil wells, and piles of compost. This group of organisms exhibit growth over a great range of temperatures and have the ability to grow well between 40 to 70 °C and optimally at 55 to 65 °C [21]; these are abnormal temperatures for most bacteria. Due to this capacity, researchers believe that the enzymes produced by the cells are also thermostable. Since *Geobacillus* species grow at much higher temperatures than *Bacillus*, it is expected that the protease from the *Geobacillus* genus will share some similar overall structures and functions to those from *B. subtilis*, though with higher stability and activity at higher temperatures.

The growth and protease production by microorganisms are greatly affected by the cultural conditions [16,22]. Hence, by manipulating the cultural conditions, the productivity of yield can be increased. Moreover, the optimization process can be also used to explore the ability of microorganisms to withstand the harsh and drastic environmental changes, which will be beneficial for production process strategies. Therefore, parameters including carbon and nitrogen sources, temperature, pH, time of incubation, NaCl concentration, and inoculum sized are often optimized to get a maximum yield of protease enzyme [16,22,23]. This present study screened, isolated, and identified a thermophilic *Geobacillus* species from Sungai Klah Hot Spring Park, Perak, Malaysia focusing on the growth dynamic of the *G. thermoglucosidasius* SKF4 and its ability to produce a thermostable protease enzyme under different cultural conditions.

## 2. Materials and Methods

### 2.1. Sample Collection

**Figure 1:**
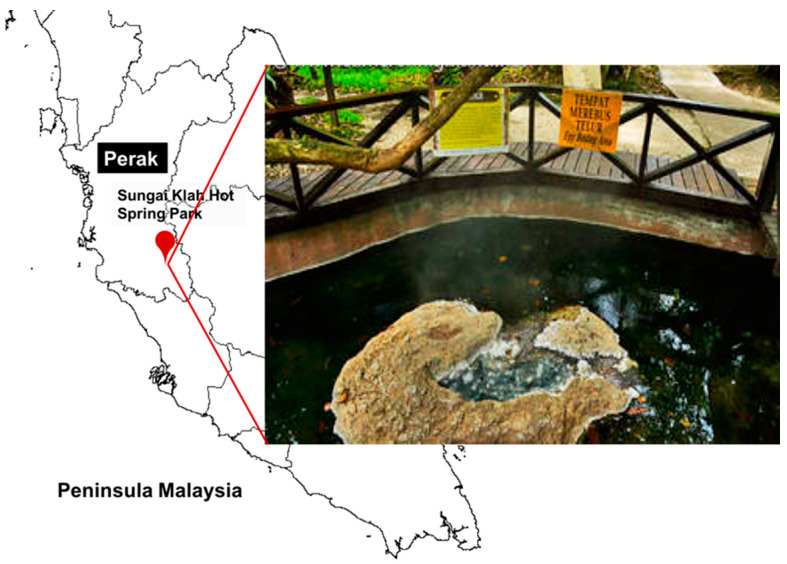
Sungai Klah Hot Spring Park located in Perak, Malaysia.

The hot spring water and soil sediments were collected from Sungai Klah Hot Spring Park located in Perak, Malaysia (03°59′40 N and 101°23′33 E) (Figure 1). According to Chan et al. [24], Sungai Klah (SK) is described as a shallow and fast-flowing stream hot spring with 150 m in length and reputed to be the second hottest hot spring in Malaysia with temperatures from 50 to 110 °C and pH of 7.0 to 9.0. Its level of total organic content (TOC) is very high relatively, due to its location in an obscure forested area and the hot spring being frequently fed by plant litter. The temperature and pH of Sungai Klah hot spring were measured in situ (89.0 to 90.5 °C and pH 8.6 to 8.7 with dissolved oxygen at 28.1%). The water samples were stored in a thermos and the soil sediments were placed in a sterilized bottle and then conveyed to the laboratory in less than 6 h.

### 2.2. Qualitative Screening of Protease Activity

The proteolytic activity of thermophilic bacteria was screened on skim milk agar (SMA). Briefly, 1 g of soil sample was added to a glass tube containing 10 mL sterilized distilled water. The soil suspension in the test tube was heated in a water bath at 80 °C for 15 min and instantly cooled in water mixed with ice. Then, 100 μL of samples were spread on SMA agar plates (1% Bactotryptone, 0.5% NaCl, 0.5% Yeast Extract and 1% Skim milk) and then incubated at 60 °C for 36 h. Colonies that produced clear zone as a result of partial hydrolysis of casein in the milk were carefully picked and re-streaked on Luria Bertani (LB) agar plate to obtain a pure culture. Additionally, the selected isolates were streaked on LB agar slants and stored in the chiller at 4 °C.

### 2.3. Protease Activity Assay

Enzyme activity was measured according to the method described by McDonald and Chen [25]. Three replicates of tubes were used for the assay; in these tubes, 2 mL of 1% (*w/v*) casein in Glycine-NaOH buffer with pH 10 was added in all three test tubes. The control contained 2 mL of 1% (*w/v*) casein solution, 1 mL of enzyme, and 3 mL 10% TCA (*w/v*). The rest of the two tubes containing 2 mL of 1% (*w/v*) casein solution and 1 mL of enzyme then were incubated at 60 °C for 15 min. After the incubation, 3 mL of 10% TCA was added and then centrifuged for three minutes. One millileter of supernatant in the test tube was added to 5 mL of alkaline copper reagent. After 15 min, 0.5 mL of Folin–Cocteau reagent was diluted in a 1:1 ratio and put to stand for 30 min. After that, the absorbance was read spectrophotometrically at 700 nm. One unit of enzyme activity is defined as the amount of enzyme that releases 1 μg of tyrosine per mL per min under the above assay conditions.

### 2.4. Morphology of the Colonies

Briefly, the isolates were grown on LB agar for 48 h and then the colony morphologies were observed with regard to color, shape, margin, internal structure, and elevation. Further, Gram staining was performed to evaluate the Gram characteristics and the cell morphologies of the isolates.

### 2.5. Molecular Identification of Isolate SKF4

The selected isolate was grown in LB broth at 60 °C overnight. After the incubation time, the cultures were centrifuged at 23,000× *g* for 5 min and the cell pellets were collected and used for DNA extraction using QIAamp DNA Mini Kit (Qiagen GmbH, Hilden, Germany) according to the manufacturer’s instructions. Further, the genomic DNA was amplified using universal primer 1492R (5′TACGGYTACCTTGTTACGACTT3′) and primer 27F (5′AGAGTTTGATCMTGGCTCAG3′). The PCR product was purified using the QIAquick PCR Purification Kit (Qiagen, Germany) and sent for sequencing at First BASE Asia Sdn. Bhd. (Selangor, Malaysia). The sequence obtained was then compared using The NCBI Basic Local Alignment Search Tools (BLAST; http://www.ncbi.nlm.nih.gov/) to search for a similar sequence with a collection of 16S rRNA sequences in the GenBank. Bacterial identifications were performed primarily based on the comparison between the physiological and microscopic characteristics outlined in Bergey’s Manual and compilation data of biochemical and morphological characteristics done on the isolates [26]. The sequences and their closest relative were retrieved and aligned with ClustalW, whereas phylogenetic tree analysis was achieved using a neighbor-joining technique by Mega software version 7.0 (The Biodesign Institute, Tempe, AZ, USA). Evolutionary distances of nucleotide sequences were computed using the Jukes–Cantor model (bootstrap values: 1000 resampling) [27].

### 2.6. Effects of Cultural Conditions on Bacterial Growth and Protease Production of Isolate SKF4

The influences of various factors on the growth and protease production of the *G. thermoglucosidasius* SKF4 were studied. Parameters such as temperature, pH, amount of sodium chloride, carbon and nitrogen supplies, and period of incubation of the bacterium were assessed following the method of Sepahy and Jabalameli [23] with some modifications. The bacterial growth was determined by measuring optical density (OD) using a spectrophotometer at 600 nm [23]. Inoculum for protease production from *G. thermoglucosidasius* SKF4 was carried out using a modified method of Sinha and Khare [28] at pH 7 in a medium that contained 5 g bactopeptone; 5 g yeast extract; 3 g NaCl; and 0.2 CaCl, 5 g KH_2_PO_4_, and 0.1 g FeSO_4_·7H_2_O dissolved in litre of distilled water. Inoculums were regularly grown in LB broth medium. Overnight cultures were centrifuged at 23,000× *g* for 15 min at 4 °C and the supernatant was used for protease production assay following the procedure described earlier [25]. The experiment was performed in triplicate. This method was applied to all the following parameters:

#### 2.6.1. Effects of Temperature 

Briefly, about 5% inoculum of isolate SKF4 was inoculated in sterilized LB broth followed by incubation at various temperatures (40, 45, 50, 55, 60, 65, 70, and 75 °C) for 24 h with 200 rpm agitation.

#### 2.6.2. Effects of Carbon Sources 

For the effects of carbon sources, the medium was prepared at pH 7 in flasks containing 1% (*w/v*) of selected carbon sources. The carbon sources used were glucose, fructose, maltose, xylose, sucrose, and lactose. The carbon source media were autoclaved at 121 °C for 15 min and cooled down prior to inoculation with 5% (*v/v*) *G. thermoglucosidasius* SKF4 and incubated at 60 °C for 24 h with 200 rpm of agitation.

#### 2.6.3. Effects of pH 

Briefly, 5% (*v/v*) inoculums of isolate SKF4 was inoculated in a sterilized broth medium set at various pH values (pH 5, 6, 7, 8, and 9) and incubated at 60 °C for 24 h with 200 rpm of agitation.

#### 2.6.4. Effects of Nitrogen Sources 

The growth media were prepared containing 1% (*w/v*) of one of the following nitrogen sources: sodium nitrate, urea, casein, ammonium sulfate, or yeast. The media were set at pH 7 and sterilized by autoclaving prior to inoculation with 5% (*v/v*) of isolate SKF4.

#### 2.6.5. Effects of Incubation Time 

An optimized production sterilized medium was prepared in a 50 mL conical flask and inoculated with 5% (*v/v*) culture of isolate SKF4. The culture media were incubated at 60 °C with 200 rpm agitation. At every 6 h interval, samples were taken and the growth and protease production were determined accordingly.

## 3. Results and Discussion

### 3.1. Isolation and Identification of Isolate SKF4

**Figure 2:**
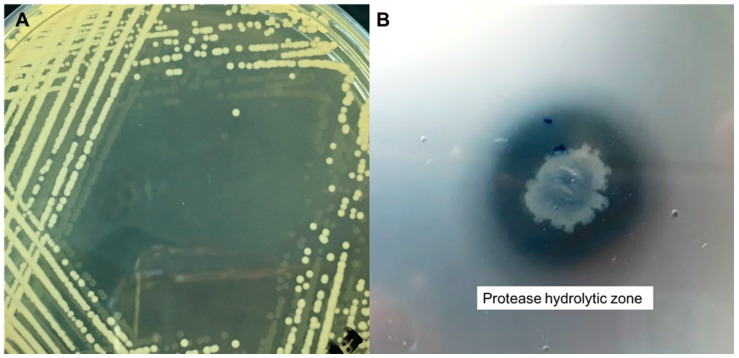
(**A**) Growth and colony morphology of isolate SKF4 on Luria Bertani agar. (**B**) Protease activity of isolate SKF4 on skim milk agar.

Investigations of hot spring microbial populations can provide an amazing and unlimited supply of new microorganisms that may perhaps display an important variation from known terrestrial phylotypes. From these organisms, we can obtain a series of enzymes and other products, which might offer a very important resource for the advancement of biotechnology. In the present study, a total number of 14 isolates belonging to a different group of bacteria were successfully isolated from the Sungai Klah Hot Spring Park located in Perak, Malaysia (Figure 1). The temperature of the Sungai Klah hot spring water was in the range of 60 to 90 °C, while the pH recorded was in the range of 8.6 to 8.7. All the isolates were observed to hydrolyze skim milk agar with the production of a clear zone around the colonies. Further, the isolates were screened both quantitatively and qualitatively for protease enzyme production (Table 1), resulting in one isolate that exhibited a promising protease production potential (175 U/mL); namely, isolate SKF4 (Figure 2B), which we selected for identification and subsequent studies. Our finding is in agreement with that by Brumm et al. [12], who reported that *Geobacillus* species could be isolated from alkaline or neutral hot springs with temperatures between 60 and 80 °C, which is an important natural environment for the growth of genus *Geobacillus*.

Based on the presence of certain physiological and phenotypical properties, such as endospore formation and its ability to grow and produce enzyme optimally at elevated temperatures, isolate SKF4 was preliminarily identified as *Geobacillus*. Morphologically, the isolate SKF4 produced large colonies with circular borders, convex in elevation with an entire margin, and identified as opaque with respect to opacity (Figure 2A). Moreover, SKF4 was found to be a Gram-positive rod-shaped bacterium. The morphological and physiological characterization and identification of SKF4 that belonged to the genus *Geobacillus* agree with the conclusions of earlier reports [29,30,31].

**Figure 3:**
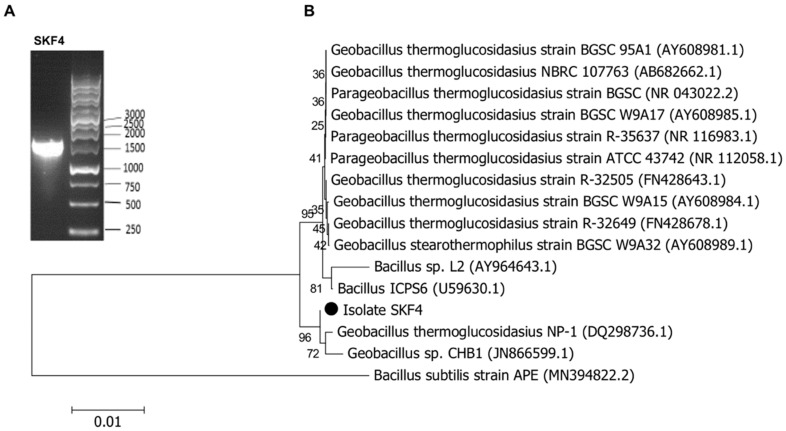
(**A**) The agarose gel electrophoresis of 16S rRNA amplified from the genomic DNA of the isolate SKF4 using 27F and 1492R primers. (**B**) Phylogenetic tree of *Geobacillus thermoglucosidasius* SKF4. The tree is drawn to scale, with branch lengths in the same units as those of the evolutionary distances used to infer the phylogenetic tree. The analysis involved 15 nucleotide sequences of some closely related species. *Bacillus subtillis* was placed as an outer group.

In this study, the amplified 16S rRNA gave an intact band of 1500 bp DNA fragment (Figure 3A). Further, the 16S rRNA gene analysis of isolate SKF4 revealed the highest (98% to 99%) sequence similarities with genus *Geobacillus* and was concluded to belong to *Geobacillus* species. Based on the phylogenetic relationship analysis using the neighbor-joining method (Figure 3B), isolate SKF4 has a very strong relationship with strain *G. thermoglucosidasius* NP-1 with 99.87% similarity. These results are in agreement with the report of Nazina et al. [8], which stated that there is more than 96% sequence homology among the members of *Geobacillus* species. Thus, the isolate SKF4 can be allocated into *G. thermoglucosidasius* considering that it has 99% similarity with the species of *G. thermoglucosidasius* and tentatively identified as *G. thermoglucosidasius* SKF4. The nucleotide sequence of the 16S rRNA gene of *G. thermoglucosidasius* SKF4 was deposited to GenBank under the accession number of MN960021.

A few chemicals and enzymes have been produced from *G. thermoglucosidasius*, mostly ethanol; however, little information about the production of serine proteases has been found. *G. thermoglucosidasius* species have been observed to be sources of many enzymes, including an alcohol dehydrogenase (ADH) enzyme for isobutanol production [20] and acrylamidase [32]. Cha and Chambliss [32] reported the cloning of the acrylamide gene from *G. thermoglucosidasius* AUT-01, a nitroalkane-oxidizing enzyme, and the cloning of the gene [33]. Additionally, a novel β-xylosidase enzyme that belongs to CAZy glycoside hydrolase family GH52 has been cloned and expressed in *Escherichia coli* [34]. Moreover, Szeker et al. [35] described the cloning and expression of two thermostable pyrimidine nucleoside phosphorylases (PyNP) isolated from *G. thermoglucosidasius* and *Thermus thermophilus*. Further, a Laccase gene from *G. thermoglucosidasius* 95A1 was successfully cloned and expressed in *E. coli* DH5α [36]. To our knowledge, no study reported the production of thermostable alkaline protease by *G. thermoglucosidasius*. Therefore, our isolate, strain SKF4 will be the first report of the production of the protease enzyme by *G. thermoglucosidasius* sp.

Furthermore, a more precise classification should be considered in the future, which will provide additional characteristics, such as DNA-DNA hybridization data. Information regarding the use of *Geobacillus* sp. to produce enzymes is rare, especially protease enzymes. However, there is no published work on the evaluation of the dynamic growth and protease enzyme production by *G. thermoglucosidasius*. To our knowledge, this is the first study on *G. thermoglucosidasius* ever conducted.

### 3.2. Effects of Different Parameters on the Growth and Protease Production of G. thermoglucosidasius SKF4

#### 3.2.1. Effects of Temperature

**Figure 4:**
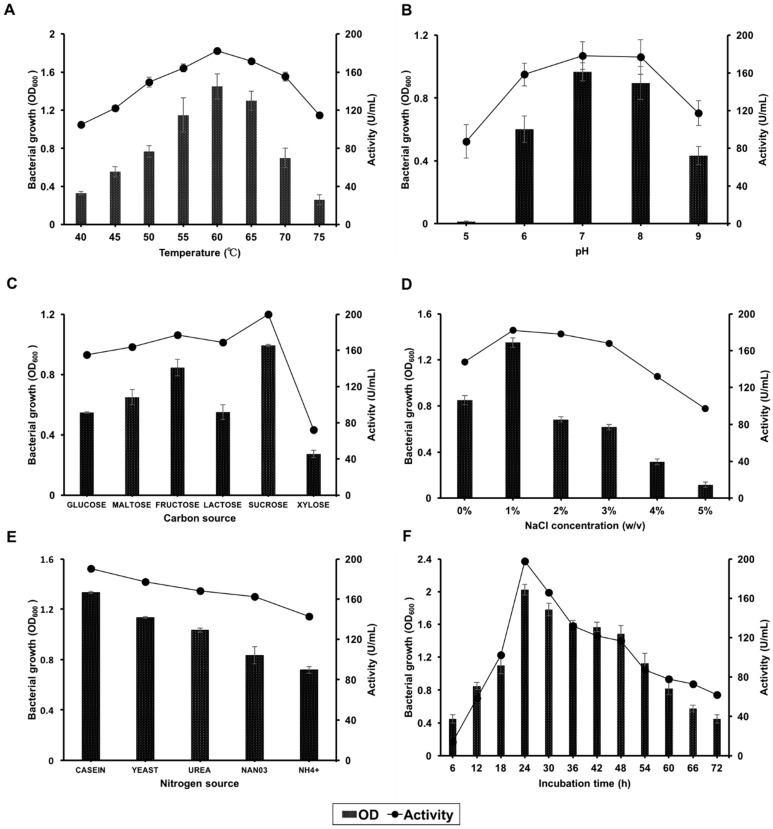
Effects of (**A**) temperature, (**B**) pH, (**C**) carbon source, (**D**) NaCl concentration, (**E**) nitrogen source, and (**F**) incubation time on growth and protease production of *G. thermoglucosidasius* SKF4. Data are expressed as means (*n* = 3) ± SDs.

Temperature has been identified as among the essential factors influencing bacteria growth and their ability to produce protease enzymes. Consequently, isolate SKF4 was grown at different temperatures (40, 50, 55, 60, 65, 70, and 75 °C). In this study, isolate SKF4 showed optimum growth at a temperature between 55 and 65 °C and maximum growth at 60 °C. Further, the production of protease enzyme occurred between the temperatures of 55 and 65 °C with maximal activity of 182 and 171 U/mL at 60 and 65 °C, respectively (Figure 4A). In previous studies, the growth and production of protease by *Geobacillus* species have been reported to fall between 55 to 70 °C. Dissanayaka and Rathnayake [37] reported the optimum growth of some *Geobacillus* sp. between 30 and 70 °C with maximum activity at 60 °C. Similarly, other reports showed the growth of thermophilic *Geobacillus* isolate producing protease enzyme at optimum temperatures ranging from 60 to 62 °C [13]. Additionally, Chen et al. [38] reported a thermophile *G. caldoproteolyticus* isolated from sewage with the capacity to grow at 35 to 65 °C. Moreover, reports by Nielsen and Villadsen, [39] and Bhunia et al. [38] stated that temperature affects the specific growth rate of bacteria; there was a gradual increase in specific growth rate until the optimum temperature and then a rapid decrease beyond the optimum temperature. However, this observation was species-dependent, as it has been reported by Shanthakumari and Boominathan [40] that there is a great variation among the optimum temperatures required for different species for protease production.

The ability of thermophilic bacteria to withstand high temperatures could be explained by the activity in their metabolic functions associated with their physiological adaptations. There are two ways in which the effects of temperatures on the organism can be explained. When temperature increases, chemical reactions and enzymatic reactions proceed faster and the growth rate also increases. Additionally, when the temperature increases, proteins become irreversibly damaged. Besides, thermophiles are much more stable to heat through molecular adaptation due to their intracellular enzymes, and other factors, such as salts, high protein concentration, coenzymes, substrates, activators, and general stabilizers [41]. Chakravorty and Petra [42] and Margaryan et al. [43] suggested that the most important mechanistic determining factors of thermoadaptation in Bacilli are the adaptation of composition regarding the membrane lipids, heat shock membrane synthesis (HSPs), and the ability of the enzyme to adapt to produce molecular stability and structural flexibility. Furthermore, thermoadaptation of the thermophiles was also due to the GC content in their genome, which was observed to be very high. In this study, isolate SKF4 demonstrated maximal protease activity at a high temperature, which is acceptable for industrial characteristics.

#### 3.2.2. Effects of pH

The changes in pH noticeably affected the growth and activity of the bacteria isolate SKF4. It was observed that the variation in the pH played a key role in the growth and protease activity of the bacteria. Highest growth and protease production were observed at pH 7 and 8 (Figure 4B). Similarly, Sepahy and Jabalameli [23] reported the maximum growth and protease production by *Bacillus* sp. at pH 8, which is in agreement with our finding on the relationship between the growth of bacteria and their production of protease enzyme. Most of the *Geobacillus* sp. reported have their growth at a pH range of 6–9 for the production of protease [38,44].

For the ability of an organism to produce protease extracellularly, the pH of the environment or culture medium is a very important condition to consider; this is due to the binding of substrate to the catalyst, and a catalytic reaction usually takes place depending on the charge distribution of substrates and enzyme molecules [45]. Additionally, the metabolic pathways of microorganisms are affected by pH. When there is an adjustment in the pH of the external medium, there is also a change in the ionization of the nutrient molecules, which consequently reduces the nutrients available to the bacteria and hence reduces the overall metabolic activity [45]. The pH of a medium influences the transportation of various constituents across the membrane. The effects of pH on bacterial metabolism in culture media, including broth, on a molecular basis are still hard to understand. The pH of a medium influences the motive force of the proton in chemiosmosis; therefore, there is a possibility that the relative efficiency of its metabolic activities is attained maximum under the optimum pH range [46].

#### 3.2.3. Effects of the Carbon Source

The influences of various carbon sources on cell growth and protease production were studied using glucose, maltose, fructose, lactose, xylose, and sucrose. In this study, isolate SKF4 showed good growth for most of the carbon sources used with the exception of xylose. Among the carbon sources, sucrose has the highest protease activity with 200 U/mL, followed by fructose with 177 U/mL. However, lower activity was recorded in glucose and xylose, 155 and 72 U/mL, respectively (Figure 4C).

The capability of an organism to break down different carbohydrates could be a result of the preference of the microorganism towards living in an environment that is low in organic content, and therefore the need to develop its systems to adjust to absorption of any food available in such environments [47]. Glucose is regularly used as a carbon source to produce protease enzymes through fermentation. Reports from different investigations showed that the production of protease enzyme was reduced by glucose due to glucose catabolic repression [48,49,50].

Moreover, other carbohydrates have been reported by many investigators to cause an increase in the yield of alkaline protease. For example, Malathi and Chakraborty [51] stated in their work that maltose is a good source of carbon for the high production of protease enzyme. Tsuchiya et al. [52] also reported the use of maltose for high production of protease enzyme, while Phadatare et al. [53] and Ibrahim et al. [1] reported on high protease enzyme activity using sucrose and fructose respectively. Further, Yang et al. [54] published that *G. thermodenitrificans* SG-01 utilizes fructose to grow, while *G. thermodenitrifcans* SG-02 utilizes fructose and glucose for maximum growth and *G. denitrificans* SG-03 utilizes sucrose.

#### 3.2.4. Effects of NaCl Concentration

The results presented in Figure 4D show that the isolate SKF4 was capable of growing over a wide range of NaCl concentrations from 0% to 5% with optimum growth and protease production activity (182 U/mL) at a concentration of 1% with appreciable activity at 2% and 3% concentrations. However, the growth of isolate SKF4 and its protease production drastically dropped when grown at 4% and 5% NaCl concentrations (Figure 4D). Similarly, Zahoor et al. [15] reported the growth of isolate *Geobacillus* TP-5 in the range of 0% to 3.5% NaCl concentration with an optimum at 0.5%. Our findings are also in line with the work of Yang et al. [54] who reported the growth of *Geobacillus* sp. SG01 at NaCl concentrations of 0% to 2% with optimum growth at a 1% concentration of salt. Likewise, *Geobacillus* sp. from other studies has been also reported having the ability to grow within the range of NaCl concentration 0% to 5% [29,55]. Contradictorily, Kalwasinska et al. [56] reported an increase in protease production as the NaCl concentration increased (1% to 9% NaCl) with maximum production at 4–5% NaCl concentration for *B. luteus* H11. The strain H11 was isolated from a highly saline soda-lime pond, and proved to be highly tolerant to salinity [56]. In this study, isolate SKF4 was isolated from a hot spring with low level of salt concentration, which might be the reason why the isolate SKF4 has optimum production at a 1% *w/v* NaCl concentration. Moderately Gram-positive halophiles usually reduce enzyme production at a high salt concentration [56,57]. Our isolate can also be considered to be moderately halotolerant.

#### 3.2.5. Effects of Nitrogen Sources

Various nitrogen sources were investigated for the growth and protease production of isolate SKF4. Results obtained (Figure 4E) revealed that the use of casein as a nitrogen source demonstrated maximum growth and protease production (190 U/mL) of isolate SKF4, followed by yeast extract. Different organisms require different specific nitrogen supplements for growth and metabolism. Our result is in line with Khusro et al. [45], who reported that the production of protease by bacteria increased when supplemented with 1% (*w/v*) of casein. Sharma et al. [58] stated that different organic nitrogen sources, either complex or simple; inorganic sources; and amino acids, have been used as good sources of nitrogen among researchers to improve the production of protease enzyme. Nitrogen sources like yeast extract have been discovered to be good sources for maximum production of protease due to various amino acids, vitamins, and carbohydrates that are enough to support growth and enhance the metabolic functions of the bacteria [59]. This is in agreement with the findings of Khusro [45], who reported that yeast extract played a crucial role in the production of the enzyme due to the presence of important elements and growth factors for microorganisms [45]. Prakasham et al. [60] reported the use of a complex organic nitrogen compound resulting in an increase of protease amount secreted compared to that when using an inorganic nitrogen compound. Similarly, Qureshi et al. [61] reported an increase in the amount of protease produced by *B. subtilis* EFRL 01 when using organic nitrogen sources from yeast extract.

#### 3.2.6. Effects of Incubation Time

In this study, the effects of incubation time (6 to 72 h) on growth and protease production of isolate SKF4 were examined. The results obtained showed that the best growth for isolate SKF4 was between 24 and 48 h with the highest growth observed at 24 h. Additionally, protease production was seen to be optimal at 24 and 30 h with 200 and 165 U/mL activity, respectively, and rapidly decreased in activity by up to 72 h at a constant rate (Figure 4F). The production of protease was observed to be proportional to the growth of the organism. This is in agreement with Thebti et al. [44], who stated that the production of protease enzyme is proportional to the bacterial growth, and that maximum growth and protease production of *G. toebii* strain LBT 77 were observed at 42 h of incubation, which also coincides with the end of stationary phase with maximum protease production of 1900 U/mL. The production of protease and maximum growth of isolate SKF4 after 24 h of incubation might be a result of the increased growth rate of the organism due to the high rate of metabolism, allowing it to reach the stationary phase at a shorter time of incubation. The finding is also in accordance with that by Chen et al. [38], who observed protease maximum production in *G. caldoproteolyticus* at late stationary growth phase with maximum protease activity after 9 h of incubation.

## 4. Conclusions

A number of protease-producing bacterial strains have been screened and isolated from water and soil sediment of Sungai Klah Hot Spring Park, Malaysia. The isolate that demonstrated the highest clear zone on skim milk agar and the highest activity of protease production was selected for identification and further investigation. Based on the 16S rRNA gene analysis, SKF4 was identified as *G. thermoglucosidasius*. The study on the effects of various parameters had showed the organism’s highest ability to produce protease enzyme and optimum growth at the high temperature of 60 and 65 °C with a pH of 7 and 8. This indicates that the organism has the capacity to produce thermostable protease. This study has also revealed that the *G. thermoglucosidasius* SKF4 was able to utilize various sugar and nitrogen sources for growth and protease production, besides showing a maximum capacity to utilize casein and yeast as sources of nitrogen and sucrose and fructose as good sources of carbon. A new alternative source of protease for commercial production of thermostable protease is required to find a better alternative for commercial use [23]. Thus, it can be stated that *G. thermoglucosidasius* SKF4 has a promising potential to be used as thermostable protease producer for commercial use in the industry with regards to its high activity at high temperature and high pH with moderate salinity. The gene encoding the thermostable protease could be cloned into a mesophilic organism like *E. coli*.

## Figures and Tables

**Table 1:** Protease activity and zone of inhibition of isolates from Sungai Klah hot spring.

Isolate	Zone of Hydrolytic Halo (cm)	Activity (U/mL)
F1	3.0	148
F2	3.0	160
F3	3.5	150
F4 ^a^	3.5	175
C1	3.0	145
C2	3.3	145
C3	3.1	140
C4	3.0	141
C5	3.2	100
C6	2.0	80
D2	2.8	75
L8	3.0	81

^a^ Indicating the strain that exhibited the highest protease activity and zone of inhibition.
